# A glimpse into the genotype and clinical importance of non tuberculous mycobacteria among pulmonary tuberculosis patients: The case of Ethiopia

**DOI:** 10.1371/journal.pone.0275159

**Published:** 2022-09-26

**Authors:** Alem Alemayehu, Abebaw Kebede, Sebsib Neway, Efrem Tesfaye, Betselot Zerihun, Melak Getu, Beyene Petros

**Affiliations:** 1 Department of Microbial, Cellular and Molecular Biology, College of Natural and Computational Sciences, Adds Ababa University, Addis Ababa, Ethiopia; 2 Armauer Hansen Research Institute, Addis Ababa, Ethiopia; 3 College of Health and Medical Sciences, School of Medial Laboratory Science, Haramaya University, Dire Dawa, Ethiopia; 4 Ethiopian Public Health Institute, Addis Ababa, Ethiopia; Shandong Public Health Clinical Center: Shandong Provincial Chest Hospital, CHINA

## Abstract

Laboratory identification of nontuberculous mycobacteria (NTM) species is not regularly performed while, they have a public health importance with a prevalence of more than 5% among pulmonary tuberculosis (PTB) patients in Ethiopia. Hence, this study aimed to identify the NTM species and their clinical significance among PTB patients. A retrospective study was conducted at the Ethiopian Public Health Institution’s (EPHI’s) national TB referral laboratory. Stored NTM isolates were genotyped using GenoType Mycobacterium CM/AS kit (Hain Life science, Germany). Data pertinent to the study was extracted from the EPHI’s database and patients’ medical records. Between January 2 & December 28 of 2017, a total of 3,834 samples were processed from 698 TB patients of whom 50% were female. Among 3,317 samples with mycobacterial culture results 7.3% were NTM and majority of them were identified from smear negative TB patients. *M*. *simiae* was the /predominant NTM among the genotyped isolates. All the studied NTM species were not clinically important however, considering the similarity of clinical and radiologic findings between NTM and MTBC infected patients, integrating NTM species identification in the routine TB laboratory diagnosis may augment clinicians’ decision particularly in DR-TB patients. Additional similar prospective study with a larger sample size is recommended. Moreover, urgent improvements on patients’ record keeping practice are required in the studied hospitals.

## Introduction

Non tuberculous mycobacteria (NTM) are environmental species of the genus *Mycobacteria* that includes the most deadly human pathogen, *Mycobacterium tuberculosis complex* (MTBC) and the disfiguring, *Mycobacterium leprae* [1]. NTM can be classified as a slow-growers including the *M avium Complex* (MAC) and *M*. *simiae*, that require more than seven days to form colonies and rapid grower like *M*. *fortuitum & M absiccuss* which require less than seven days to form colonies [2]. Majority of the NTM are recovered from environmental sources including water and soil. Unlike other mycobacterial infections, human to human transmission of NTM is not reported. Hence, human infection is acquired from environmental exposures [3]., However, whole genome sequencing and epidemiological studies have been reported the transmission ability of NTM among cystic fibrosis patients particularly, *M*. *absiccuss and* MAC species [4,5].

NTMs are becoming epidemiologically important since their clinical significance is increasingly recognised [6,7]. In Western countries, MAC [8,9] and in parts of Africa, *M*. *kansasi*, *and M*. *abscesses* [10] have been the most common NTM species that are responsible for pulmonary infections. Unlike in developed countries, the distribution of NTM and the prevalence of their disease are not fully understood in the developing countries, including Ethiopia.

The similarity in signs and symptoms of pulmonary diseases caused by NTM and MTBC actually need accurate clinical and laboratory diagnosis as well as, different drug treatment as indicated in the American Thoracic Society or the Infectious Diseases Society of America (ATS/IDSA) diagnostic guideline for NTM infection [11]. However, the major TB laboratory diagnostic tool are smear microscopy; less sensitive and GeneXpert: that could not detect NTM and also not fully implemented in Ethiopia yet. These all-causes difficulty in diagnosis and treatment of NTM infection particularly in smear negative PTB patients, that have been diagnosed based on clinical and image findings, in countries like Ethiopia with a limited laboratory infrastructure and Mycobacteria detection facilities including culture and molecular tests.

During a routine culture isolation of MTBC, most Ethiopian mycobacterial laboratories have been reporting NTM without further characterization to species level which is important for determining their clinical relevance to exclude their contaminant role as well as NTM disease management which is species specific [11]. Limited studies have shown the occurrence and species of NTM among clinical samples [12–14] but there is no documented data on their involvement in clinical disease in the country. Therefore, the current study tried to identify the NTM species and their clinical significance in PTB patients and it also described the NTM distribution with respect to demographic and some other clinical data of PTB patients.

## Methodology

A cross- sectional retrospective study was conducted on PTB patients whom, their MTB culture result were available from January 2, 2017 to December 28, 2017 in EPHI TB database. EPHI is a national TB reference laboratory of Ethiopia that is located in the capital Addis Ababa. Similarly, NTM species identification study was conducted using stored culture grown isolates available during the study period. Socio-demographic, clinical and laboratory data were extracted from the EPHI’s database and the patients’ medical record.

Subsequently, based on the recorded address of referring health facility from the EPHI database NTM identified patients who were diagnosed in hospitals located in Addis Ababa were included irrespective of the drug resistance profile for further NTM clinical importance study. Patients ‘medical record were critically revised using a structured data collection format which includes demographic, clinical and image findings as well as other comorbidities. Subsequently, clinical significance of the identified NTM species were determined based on the ATS/ IDSA guideline [11] that includes both clinical and microbiologic findings.

### Culture identification

Respiratory samples of PTB patients that were referred to EPHI either for the diagnosis or follow up purpose were subjected to culture and acid-fast staining (AFS) according to the routine mycobacteriology laboratory analysis for TB diagnosis and MTBC isolation.

Briefly, sputum samples were treated with N-acetyl-L-cysteine-sodium hydroxide and inoculated on Lowenstein Jensen (LJ) TB culture media. Portion of the processed samples were used for Ziehl Neelsen (ZN) staining smear microscopy analysis. Capilia TB- Neo test (TAUNS Laboratories Inc, Numazu Japan) was done for LJ culture grown isolates to differentiate whether the isolate is MTBC or NTM [15]. All the laboratory analysis were performed based on the national guidelines for TB laboratory diagnosis [16] and the standard operational (SOP) of EPHI.

### Genotyping of the NTM

Species identification was done from stored NTM isolates using Genotype CM Ver 2.0 and Genotype AS Ver 1.0 (Hain Life science, Germany) test kits. Initially, DNA extraction was performed using heat and centrifugation after thawing and reconstructing the stored NTM isolates strictly following SOP of EPHI.

DNA amplification was performed accordingly using reagents supplied with the GenoType mycobacteria CM/ AS assay kit using a PCR thermocycler (Applied Biosystems Model 2720, USA). Hybridization and detection procedure were performed manually using water bath with shaker. Finally, NTM species were interpreted and decided following the manufacturer instruction. All the procedures were performed according to the SOP and manufacturer instructions(https://www.immunodiagnostic.fi/wp-content/uploads/GenoType-CM-V2_kit-insert.pdf & https://www.immunodiagnostic.fi/wp-content/uploads/GenoType-AS_kit-insert.pdf).

For operational reason, pulmonary TB patients were defined as TB patients diagnosed based on the previous national guide line [16] irrespective of TB treatment history (new, relapse, treatment failure and follow up. Similarly, clinically significant NTM were defined as NTM species identified from PTB patients and fulfil the ATS/ IDASA NTM diagnosis criteria [11].

### Statistical analysis

Data for variable of interest were exported from the database to Microsoft excel, checked and cleared for further statical analysis. Microsoft excel and SPSS version 25 applications were used for the analysis and data presentation. Mean ±SD, Frequency and percentage were used to describe the data.

### Ethical considerations

The study was evaluated and approved by the Institutional Research Ethics Board of Addis Ababa University, College of Natural Science and Ethics committee of Addis Ababa city administration health bureau. The IRB or ethics committee waived the requirement for informed consent since the study was a retrospective. Official latter of co-operation was written for EPHI and respective health institutions and permission was obtained. Confidentiality of patient’s data was maintained.

## Results

From January 2, 2017 to December 28, 2017, a total of 3,834 samples were processed from 698 TB patients (50.6% female and 49.4% male) with a mean age of 32.4 (±11 SD) years. Of the total, 505 (76.3%) patients had a record of their address and majority of them were from Addis Ababa City Administration 312(63.4%) and the remaining 193 patients were from the different regions of the country; Tigray (19%), Oromia (11%), Southern Nations Nationalities and Peoples (SNNP) (2.4%) and the rest accounting for the 4.2%.

**Fig 1:**
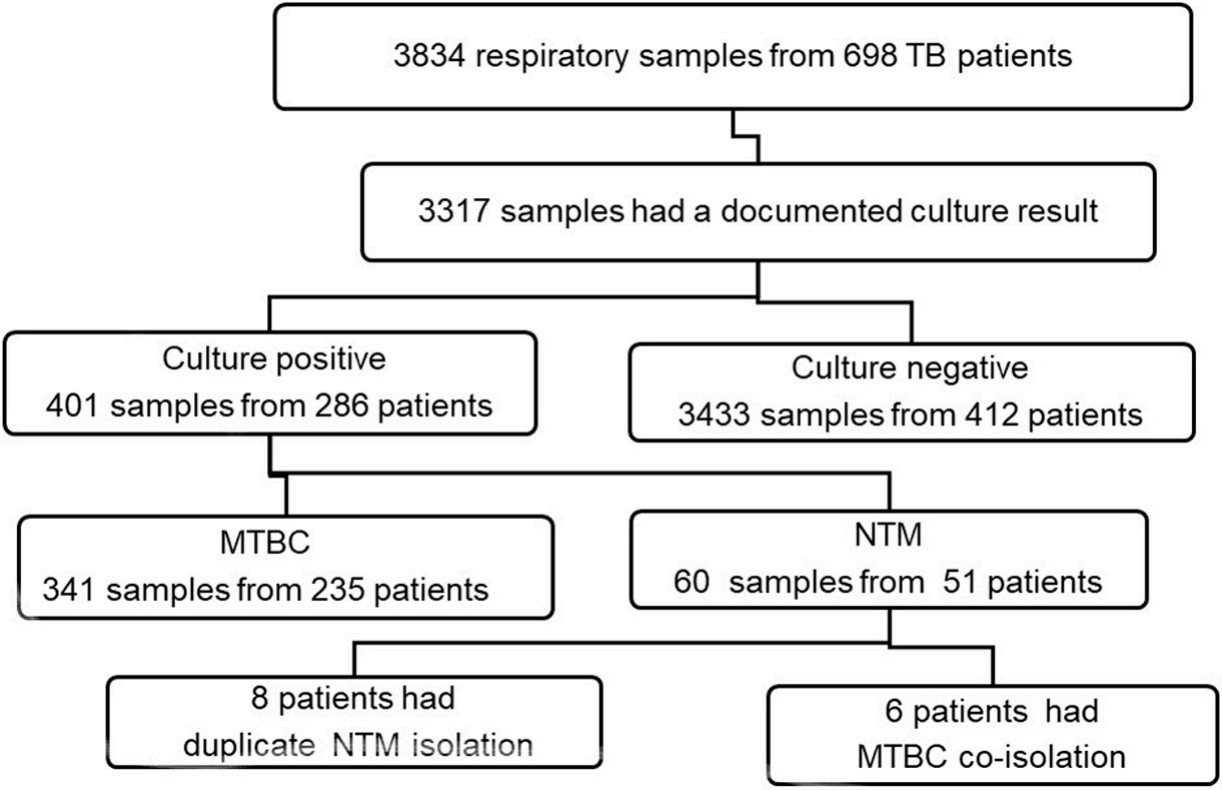
Flow chart of PTB patient and sample identification with NTM and MTBC.

The mycobacterial culture result of 3317(86.5%) samples were documented, and NTM was isolated from 51/697(7.3%) patients and among these 6 patients had MTBC co-isolation and 8 patients had a duplicate or more isolation of NTM from their samples as presented on Fig 1. Only one patient had extra pulmonary TB and the remaining 50 were PTB (S1 Table).

The frequency of NTM isolation was 29.4% among patients with age group 20–29 years followed by 30–39 years 25.5% without gender difference as shown in Table 1. Out of the 51 patients 23 (45%) had a record of HIV test result and only eight (34.5%) had reactive HIV test result. Of the t 34 patients who had a record of TB classification, 27.4% were new case and 7/51 (13.7%) were treatment frailer. Significantly, higher number of NTM were recovered from AFB smear negative patients (82.4%) of whom 39 (92.8%) were tested for follow up purpose.

**Table 1:** Frequency distribution NTM isolation among PTB patients at EPHI Ethiopia.

Study variables		Frequency	Percent (%)
**Gender**	Women	26	51
	Men	25	49
	NR	2	3.9
	Total	51	100
**Age group**	</ = 19	10	19.6
	20–29	15	29.4
	30–39	13	25.5
	40–49	5	9.8
	50–59	3	5.9
	>/ = 60	3	5.9
	Total	51	100
**HIV status**	Non-reactive	15	29.4
	Reactive	8	15.7
	NR	28	54.9
	Total	51	100
**TB classification**	New	14	27.5
	Relapse	12	23.5
	Default	1	2
	Treatment failure	7	13.7
	NR	17	33.3
	Total	51	100
**Reason for test**	Diagnosis	6	11.8
	Follow up*	39	76.5
	NR	6	11.8
	Total	51	100
**AFB smear result**	Negative	42	82.4
	Positive	9	17.6
	Total	51	100

*Includes all patients referred for MTBC culture after starting anti TB drug treatment irrespective of the follow up month, NR: Not recorded.

### Species of NTM

A total of 71 stored NTM isolates were involved for species identification study including 21 isolates from patients diagnosed in hospitals found in Addis Ababa and 50 isolates recovered from referral samples sent from different regions of the country. Among the 71 isolates, 25 had a valid positive result and 18 of them were NTM and 7 were MTBC with the Genotype CM assay. Whereas the remaining 28 isolates had a negative and 18 isolates had invalid result.

In a subsequent analysis of the 46 isolates which had either negative/ invalid result with Genotype Mycobacteria CM assay, 21 had a valid result with Genotype Mycobacteria AS assay. Among these 17 were positive for NTM, 3 were MTBC, and 1 isolate had a negative result. Overall, among the 71 genotyped probable NTM isolates only 49.3% (35/71) NTM and 14.1% (10/71) were MTBC and the remaining 1 sample had a negative result 25(35.21%) samples had invalid by both the Genotype mycobacterial CM/AS assay.

**Fig 2:**
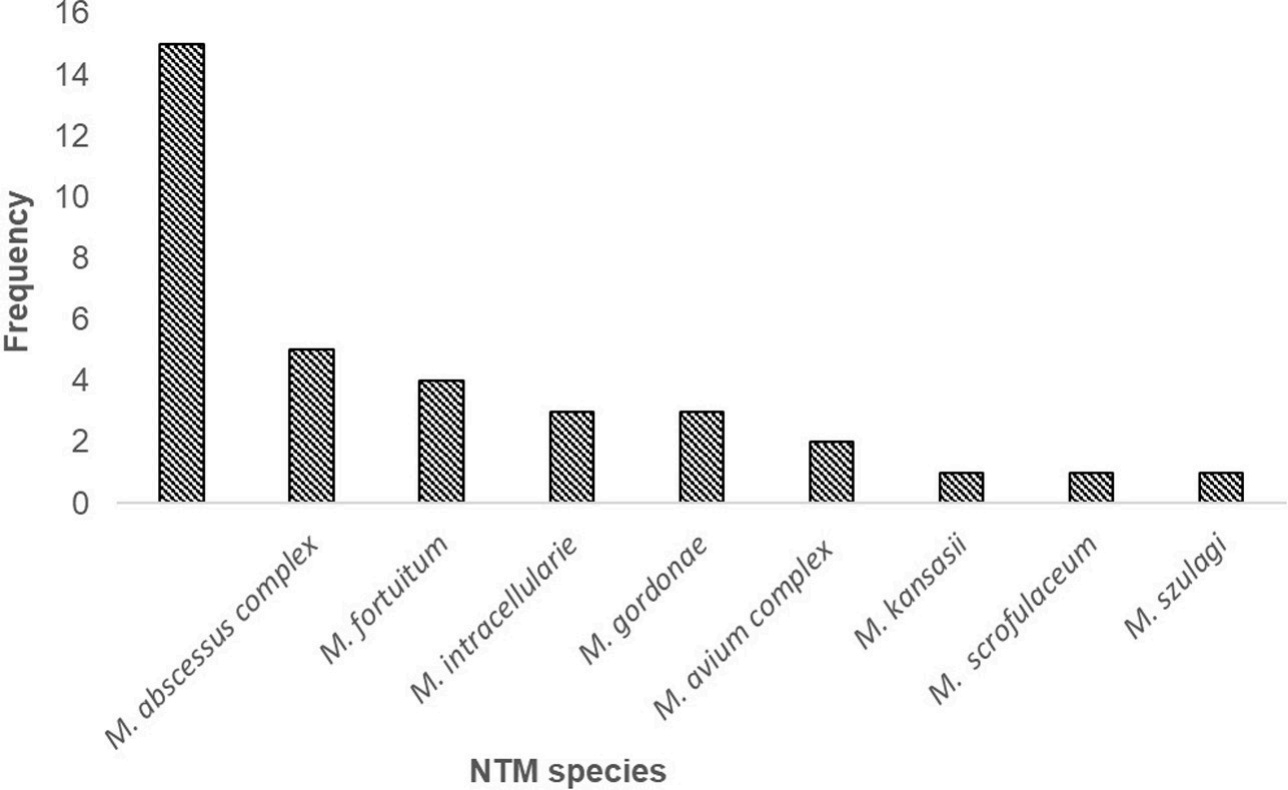
NTM species isolated from PTB patients at EPHI, Ethiopia.

The NTM genotyped in this study were grouped in to nine species, 7 from slow grower and 2 from rapid grower. *M simiae* 43% (15/35) was the most frequently isolated slow grower NTM species followed by *M abscessus* 14% (5/35) a rapid grower NTM species as shown in Fig 2.

### Clinical significance of NTM

Fifteen patients (15/35) whom NTM species were identified from their sputum sample and got treated for PTB in hospitals located in Addis Ababa city were involved for the NTM clinical significance assessment. The medical record of only 6/15(40%) patients were available and reviewed accordingly. Of these 5 of them were follow up cases and also treated for multi drug resistant TB (MDR-TB) as shown in Table 2. Whereas the medical record of 5 patients were not allowed to be reviewed because of their involvement in another clinical trial study performed by WHO and medical record of 4 patients were not found.

**Table 2:** Patients clinical and microbiologic characteristics according to the AST diagnostic guide line.

Patient Ser No	NTM species	AFB	TB classification	Treatment line	Underlying comorbidities	Clinical (All required)			Microbiologic			Remark
						Clinical: Pulmonary symptoms	Radiography: nodular or cavitary opacities or multifocal bronchiectasis with multiple small nodules	Appropriate exclusion of other diagnoses	Positive culture results from at least two separate expectorated sputum samples	Positive culture result from at least one bronchial wash or lavage	Transbronchial or other lung biopsy with mycobacterial histopathologic features and positive culture for NTM or biopsy showing mycobacterial histopathologic features	
**1**	*M scrofulaceum*	Negative	New case	2nd line	NR	Cough, Chest pain, weight loss	NR	Improved with anti- TB drug treatment	Only from single sputum	ND	ND	**CNI**
**2**	*M szulgai*	Negative	Treatment failure	1st&2nd line		NR	NR	cured with anti-TB drug treatment	Only from single sputum	ND	ND	**CNI**
**3**	*M abscessus*	Negative	New case	2nd line	DMII	Cough, weight loss	NR	Improved with anti- TB drug treatment	Only from single sputum	ND	ND	**CNI**
**4**	*M fortuitum*	Negative	Treatment failure	1st&2nd line	NR	Cough, chest pain	NR	NR	Only from single sputum	ND	ND	**ND**
**5**	*M simiae*	Negative	Treatment failure	2nd line	NR	Cough, chest pain, fever, weight loss, fatigue	NR	Improved with anti -TB drug treatment	Only from single sputum	ND	ND	**CNI**
**6**	*M simiae*	Positive	Relapse	NR	NR	Cough, chest pain, fatigue	NR	NR	Only from single sputum	ND	ND	**ND**

NR; Not recorded, CNI; Clinically not important based on the stated criteria of ATS.

Based on the ATS/IDSA NTM disease diagnostic criteria NTM species identified from four patients were not found to be clinically important. Because, these patients had a favourable anti-TB drug treatment outcome and NTM was identified only from a single clinical sample. The importance of the NTM was not determined in two patients, since they did not fulfil the minimum criteria of AST/IDSA. The clinical and microbiological information of the patients are shown in Table 2.

## Discussion

The current study aimed to elucidate the frequency and types of NTM species isolated from PTB patients and their clinical importance in which published data is very scars, especially in Ethiopia. The NTM prevalence among TB patients was found to be 7.3% which is similar with a previous study from the same institution (6%) [17] but lower than recent study reported from St. Paul’s Hospital Millennium Medical College, A.A, Ethiopia, 10.3% [13], Tanzania, 9.7% [18] and India, 8.6% [19]. The discordance in the magnitude of isolation from Ethiopian study may be due to the difference in the study period, population, (PTB patient versus PTB suspects) and country versus city. However, more than 5% prevalence findings of all these studies, may show that the increasing occurrence of NTM isolation among TB patients in addition to its public health importance in Ethiopia and other developing countries.

A higher frequency of NTM was found in younger age group without gender and HIV status difference, which is different from other studies. The proportion of NTM were higher among older age group and male gender in a study report from India [19]. Similarly, the frequency of NTM isolation among HIV positive patients were higher in Tanzania, Ghana, and Zambia [18,20,21]. The difference could be due to study design applied in the current study in which the primary purpose of the collected data was detecting TB that was characterized by the dominancy of younger age group [22]. However, it is known that HIV and older age group compromises host immune status and make individuals more susceptible to NTM infection. Unlike women, men’s’ frequent exposure to outdoor activities may increase their exposure to NTM, which primarily recovered from the environment hence given the name ‘environmental mycobacteria’ [23].

Majority of NTM were recovered from TB patients with AFB smear negative result, which is in agreement with reports from Ethiopian TB prevalence survey [24]. However majority of study population in the current study were follow up cases started anti-TB drug treatment which may eradicate the TB bacilli and resulted a negative smear result. Therefore, the source of culture grown NTM recovered from smear negative patients might be a contamination from the environment since, *M*. *simiae* the most frequent species in this study which is primely found in water [11]. Otherwise, the smear negativity might be due to the lower sensitivity of AFS for mycobacteria detection plus the strong decolourizing reagent used for the staining procedure which couldn’t resisted by majority of rapidly growing NTM species may contribute for the negative smear microscopy result [25].

MAC was the first most frequently isolated NTM in majority of studies conducted in both developed and developing countries, Africa [9]. Whereas, in the current study *M*. *simiae* was the predominant isolate. This is different from other study reports from Ethiopia, Adama, MAC [14]; Addis Ababa, *M*. *peregrinum* [13], Jimma [26] and Kenya *M. fortuitum [27],* India; *M*. *avium* [28] and Gabon; *M*. *intracellulare* [29] were the predominant NTM species. However, it is similar with studies reported from Pakistan & Iran [30,31].

It is obvious that, NTM are not communicable contrasting to MTBC and their distribution varies with respect to time, geographical location and the studied population [23]. For instance, Ethiopian (Jimma) and Kenyan studies were conducted on infants and both found *M*. *fortuitum* as the most frequent species. On the other hand, *M*. *simiae* the predominant NTM in the current study may perhaps becoming a growing NTM recovered from adult PTB patients similar with the ongoing reports from developing world including Asian countries in recent years [31]. In support of this argument, *M*. *simiae* was isolated from MDR TB patient putted on second line anti TB drug treatment in the current study. Emphasizing that the similarity of *M*.*simiae* pulmonary infection with PTB clinical presentation, radiologic findings plus its culture growth characteristics.

*M*. *absiccuss complex* was the second most frequently isolated NTM species in this study which was different from the study reports from Ethiopia [13] and India that was *M. intracellulare [28],* Kenya *M*. *scrofulaceum* [27] and Iran *M. fortuitum [31]* but similar with a study report from China [32]. This variation could be due to the difference in the study population: suspects in Indian study vs patient in the current and the other study from China [32]. Nevertheless, it is well understood that *M*. *absiccuss* is the most frequent RGM NTM (80%) isolated from pulmonary samples and associated with clinical diseases [11]. Beside it is the only NTM that had the transmissible ability among cystic fibrosis patients other than MAC [4,5].

*M*. *fortuitum* was the third most frequent isolate in this study, which is different from studies conducted in Ethiopia Adama, *M*. *absiccuss*[14] and *M*. *kansasi* in China and Iran [31,32]. Similarly, the above-mentioned factors may contribute for the variation between the study reports. Nonetheless, *M*. *fortuitum* was the predominant NTM isolate from pulmonary samples from infants [26,27] that may need critical assessment on its clinical importance if it is recovered from in this group of population.

For patients who had clinical and microbiologic information, clinical importance of the identified NTM species were determined based on ATS/IDSA NTM diagnosis criteria. Thus, none of the isolated NTM species were clinically important after critical assessment of individual patients accordingly. This may emphasize that MTBC is the primary pathogen yet in patients with pulmonary symptoms in Ethiopia as evidenced by the favourable ant- TB drug treatment outcome among aa the study participants. Otherwise, it could be due to the lower sample size and retrospective study design that limited the necessary information required to evaluate the identified NTM species.

However, different study reports confirmed that, all of the clinically evaluated NTM species *M*. *abscessus*, *M*. *fortuitum* [33], *M*. *simiae*, *and M*. *szulgai* have been involved in pulmonary infections particularly in immunocompromised individuals except *M*. *abscessus M*. *fortuitum* which may cause infection irrespective of individuals immune status [34–37].

The cautious finding of this study was majority of the patients were treated for MDRTB and a recent study report showed that how strong was the clinical, radiological and laboratory findings similarities between MDR and NTM pulmonary infections [38] and NTM patients were treated as MDR TB [39]. Indicating the need for definite diagnosis PTB confirmed by laboratory tests including species identification of the isolated NTM. Unfortunately, clinical significance of the identified NTM was assessed only for 4 patients out of the 35 whereas the remaining 31 patients medical record were not accessible and/or available in the current study.

Genotyping of NTM from larger sample size, and the clinical importance study in PTB patients may be for the first time in the study area, which can be used as a base line for future studies, could be some of the strengths of the current study. On the other hand, the retrospective nature of the current study, hindered majority of the patients identified with NTM to be included in the clinical importance study. This may obscure the actual occurrence of NTM clinical disease in the study area. Similarly, the genotyping of NTM directly from stored isolates collected in routine MTBC culture procedure and the genotyping test less sensitive for some NTM species, may lowered the reported frequency and species of NTM isolated among PTB patients.

## Conclusion

A significant number of NTM were identified among PTB patients and *M*. *simie* was the predominant species in the current study. None of the NTM species assessed for clinical significance were important in this study however, additional prospective study with larger sample size in order to get the full picture of NTM infection in the study area is recommended. In the current study, poor recording and record keeping practices were observed which need urgent measures particularly documenting all TB related clinical, laboratory and radiographic findings in the studied hospitals in order to utilize for future studies like this.

## Supporting information

S1 Table Characterstics of patients identified with NTM. (DOCX)
