# Supplementary material for: A glimpse into the genotype and clinical importance of non tuberculous mycobacteria among pulmonary tuberculosis patients: The case of Ethiopia
Source: PLoS One. 2022 Sep 26;17(9):e0275159. doi: 10.1371/journal.pone.0275159 (PMC9512186; doi:10.1371/journal.pone.0275159)
Supplement: S1 Table — (DOCX) [file pone.0275159.s001.docx]

| **Serial No** | **Age** | **Sex** | **HIV Status** | **TB classification** | **Previous Treatment** | **Reason for test** | **Follow-up Month** | **Specimen Type** | **Specimen Volume** | **AFB Smear Result** | **Reported Culture Result** |
| --- | --- | --- | --- | --- | --- | --- | --- | --- | --- | --- | --- |
| NP001 | 40 | Female | None Reactive | New case | NR | Follow up | 3 | Sputum | 7 | Negative | NTM |
| NP002 | 20 | Female | NR | NR | First line | Follow up | 5 | Sputum |  | Negative | NTM |
| NP002 | 38 | Female | Reactive | NR | NR | Follow up | 15 | Sputum | 3 | Negative | NTM |
| NP003 | 36 | Female | Unknown | Relapse case | NR | NR | NR | Sputum | 3 | Negative | NTM |
| NP004 | 30 | Male | NR | NR | First line | Diagnosis | NR | Sputum | 10 | Negative | NTM |
| NP005 | 35 | Female | Reactive | NR | NR | Follow up | 16 | Sputum | 3 | Negative | NTM |
| NP006 | 84 | Male | NR | New case | NR | Follow up | 2 | Sputum |  | scanty | NTM |
| NP007 | 18 | Female | None Reactive | New case | second line | Follow up | 15 | Sputum | 2 | Negative | NTM |
| NP008 | 25 | Female | Unknown | Relapse case | First line | Follow up | 15 | Sputum | 4 | Negative | NTM |
| NP009 | 20 | Female | NR | Treatment failure | second line | Follow up | 7 | Sputum |  | Negative | NTM |
| NP010 | 18 | Female | NR | New case |  | Follow up | 5 | Sputum |  | Negative | NTM |
| NP011 | 14 | Male | Reactive | Treatment failure | second line | Follow up | 2 | Sputum | 4 | Positive +2 | NTM |
| NP012 | 25 | Female | None Reactive | NR | NR | Follow up | 12 | Sputum | 4 | Negative | NTM |
| NP013 | 28 | Male | NR | Treatment failure | First line | Follow up | 0 | Sputum |  | Positive +2 | NTM |
| NP013 |  | Male | NR | New case | second line | Follow up | 5 | Sputum | 4 | Negative | NTM |
| NP014 | 9 | Female | NR |  | second line | Follow up | 15 | Sputum |  | Negative | NTM |
| NP015 | 22 | Male | None Reactive | New case | First line | Follow up | 0 | Sputum | 4 | Negative | NTM |
| NP016 | 54 | Male | None Reactive |  | First line |  | NR | Sputum | 4 | Positive +3 | NTM |
| NP016 | 55 | Male | None Reactive | Treatment failure | New | Follow up | 14 | Sputum | 7 | Positive +2 | NTM |
| NP016 | 55 | Male | NR | New case | First line | NR | 6 | Sputum |  | Positive +3 | NTM |
| NP016 | 55 | Male | Unknown |  | NR | NR | NR | Sputum | 4 | Positive +1 | NTM |
| NP017 |  | Female | None Reactive | New case | First line | Follow up | 3 | Sputum | 4 | Negative | NTM |
| NP018 | 30 | Male | Unknown | Relapse case | second line | Diagnosis | NR | Sputum | 7 | Negative | NTM |
| NP019 | 22 | Female | None Reactive |  |  | Follow up | 5 | Sputum | 4 | Negative | NTM |
| NP019 | 22 | Female | NR | NR | First line | Follow up | 7 | Sputum | 3 | Negative | NTM |
| NP020 | 28 | Male | Reactive | New case |  | Follow up | 2 | Sputum | 4 | Negative | NTM |
| NP021 | 26 | Female | Reactive | Treatment failure | First line | Follow up | 13 | Sputum | 2 | Negative | NTM |
| NP022 | 25 | Male | NR | NR | NR | Follow up | 17 | Sputum | 5 | Negative | NTM |
| NP023 | 30 | Female | Reactive | Relapse case | second line | Follow up | 22 | Sputum | 5 | Negative | NTM |
| NP024 | 21 | Male | Unknown | Treatment failure | First line | Diagnosis | NR | Sputum | 3 | Negative | NTM |
| NP025 | 41 | Male | NR | NR | New |  |  | Sputum | 4 | Negative | NTM |
| NP026 | 20 | Female | Unknown | NR | First line | Follow up | 3 | Sputum | 3 | Negative | NTM |
| NP027 | 38 | Female | Unknown | Return after dafault | First line | Follow up | 11 | Sputum | 5 | Negative | NTM |
| NP028 | 24 | Female | Reactive | New case |  | Follow up | 0 | Sputum | 3 | Negative | NTM |
| NP029 | 23 | Female | None Reactive | New case | New | Follow up | 15 | Sputum | 7 | Negative | NTM |
| NP030 | 42 | Male | Unknown | Relapse case | First line | Follow up | 13 | Sputum | 3 | Negative | NTM |
| NP030 | 43 | Male |  | Relapse case | First line | Follow up | 7 | Sputum | 1 | Negative | NTM |
| NP031 |  | Male | None Reactive | New case | NR | Follow up | 20 | Sputum | 4 | Negative | NTM |
| NP032 | 32 | Female | Unknown | Relapse case | First line | Follow up | 21 | Sputum | 4 | Negative | NTM |
| NP033 | 60 | Male | None Reactive | New case | First line | Diagnosis | NR | Sputum | 5 | Scanty | NTM |
| NP4 | 70 | Male | Unknown | New case | First line | Follow up | 4 | Sputum | 7.5 | Negative | NTM |
| NP035 | 22 | Male | NR | NR | NR | Follow up | 19 | Sputum | 4 | Negative | NTM |
| NP036 |  | Male | None Reactive | Relapse case | second line | Follow up | 2 | Sputum | 4 | Negative | NTM |
| NP036 | 15 | Male | None Reactive | NR | First line | Follow up | 5 | Sputum | 3 | Negative | NTM |
| NP037 | 30 | Male | None Reactive | Relapse case | First line | Follow up | 7 | Sputum | 4 | Negative | NTM |
| NP038 | 50 | Female | NR | Relapse case | second line | Follow up | 15 | Sputum | 6 | Negative | NTM |
| NP039 | 49 | Male | Reactive | Relapse case | First line |  | 0 | Sputum | 3 | Positive +1 | NTM |
| NP040 | 52 | Female | None Reactive |  | NR | NR | 0 | Sputum | 5 | Positive +1 | NTM |
| NP041 | 41 | Male | Unknown | New case | First line | NR | NR | Sputum | 5 | Negative | NTM |
| NP042 | 31 | Male | NR | New case | NR | Diagnosis | NR | Sputum |  | Positive +2 | NTM |
| NP043 | 38 | Male | Reactive | NR | First line | Follow up | 10 | Sputum | 7.5 | Negative | NTM |
| NP044 | 13 | Female | None Reactive | NR |  | Follow up | 0 | Sputum | 4 | Positive +2 | NTM |
| NP045 | 5 | Male | None Reactive | Relapse case | second line | Follow up | 5 | Sputum | 3 | Negative | NTM |
| NP045 | 5 | Male | None Reactive | Relapse case | second line | Follow up | 6 | Sputum | 4 | Negative | NTM |
| NP046 | 30 | Male | NR | Relapse case | second line | Follow up | 3 | Sputum | 4 | Negative | NTM |
| NP047 | 32 | Male | NR | Treatment failure | NR | Follow up | 17 | Sputum |  | Negative | NTM |
| NP048 | 14 | Female | Unknown | New case | NR | Follow up | 18 | Sputum | 4 | Negative | NTM |
| NP049 | 25 | Male | NR | NR | First line | Follow up | NR | Sputum |  | Negative | NTM |
| NP050 | 12 | NR | NR | NR |  | Follow up | 24 | Sputum | 4 | Negative | NTM |
| NP051 | 28 | Male | Reactive | Relapse case | second line | Follow up | 4 | Sputum | 5 | Negative | NTM |

**NR: Not recorded**
